# Period prevalence and identification challenges of viral haemorrhagic fever suspect cases in a tertiary referral hospital in Guinea: a cross-sectional, retrospective study of triage and emergency room patient profiles

**DOI:** 10.1186/s12879-020-05573-8

**Published:** 2020-11-12

**Authors:** Manuel Raab, Lisa M. Pfadenhauer, Vinh-Kim Nguyen, Dansira Doumbouya, Michael Hoelscher, Guenter Froeschl

**Affiliations:** 1 Division of Infectious Diseases and Tropical Medicine, University Hospital (LMU), Leopoldstr. 5, 80802 Munich, Germany; 2 Institute of Medical Informatics, Biometry and Epidemiology, Pettenkofer School of Public Health, Ludwig Maximilian University Munich, Marchioninistr, 15, 81377 Munich, Germany; 3 Department of Anthropology and Sociology, The Graduate Institute (IHEID), Rue Eugene-Rigot 2, Case postale 1672, 1211 Geneva 1, Switzerland; 4 Paediatric Service, Hôpital Régional de N’zérékoré, Nzérékoré, Guinea

**Keywords:** Viral Haemorrhagic fever, Screening, Ebola, West Africa, Guinea, Tertiary hospital, Infection prevention and control

## Abstract

**Background:**

A functioning Viral Haemorrhagic Fever (VHF) surveillance system in countries at risk for outbreaks can reduce early transmission in case of an outbreak. Surveillance performance depends on the application of suspect case definitions in daily clinical practice. Recommended suspect case criteria during outbreaks are designed for high sensitivity and include general symptoms, pyrexia, haemorrhage, epidemiological link and unexplained death in patients. Non-outbreak criteria are narrower, relying on the persistence of fever and the presence of haemorrhagic signs.

**Methods:**

This study ascertains VHF suspect case prevalence based on outbreak and non-outbreak criteria in a Guinean regional hospital for a period of three months. The study further describes clinical trajectories of patients who meet non-outbreak VHF suspect case criteria in order to discuss challenges in their identification. We used cross-sectional data collection at triage and emergency room to record demographic and clinical data of all admitted patients during the study period. For the follow-up study with description of diagnostic trajectories of VHF suspect cases, we used retrospective chart review.

**Results:**

The most common symptoms of all patients upon admission were fever, tiredness/weakness and abdominal pain. 686 patients met EVD outbreak criteria, ten adult patients and two paediatric patients met study-specific non-outbreak VHF suspect case criteria. None of the suspect cases was treated as VHF suspect case and none tested positive for malaria upon admission. Their most frequent discharge diagnosis was unspecific gastrointestinal infection. The most common diagnostic measures were haemoglobin level and glycaemia for both adults and for children; of the requested examinations for hospitalized suspect cases, 36% were not executed or obtained. Half of those patients self-discharged against medical advice.

**Conclusions:**

Our study shows that the number of VHF suspect cases may vary greatly depending on which suspect case criteria are applied. Identification of VHF suspect cases seems challenging in clinical practice. We suggest that this may be due to the low use of laboratory diagnostics to support certain diagnoses and the non-application of VHF suspect case definitions in clinical practice. Future VHF suspect case management should aim to tackle such challenges in comparable hospital settings.

## Background

The 2013–2016 West African outbreak of Ebola Virus Disease (EVD), a Viral Haemorrhagic Fever (VHF) caused by a virus from the Filoviridae family, evolved into the largest recorded VHF epidemic in history. More than 15,000 cases were laboratory confirmed and 11,325 patients died from Ebola virus infection [1]. The outbreak most likely originated in the administrative region of N’zérékoré in *Guinée Forestière* (Forest Guinea) in December 2013 [2]. The impaired health system and the absence of VHF surveillance in the region allowed the uncontrolled spread of the virus to Sierra Leone and Liberia before *zaire ebolavirus* was confirmed as causative agent in March 2014 [3]. This delay between the occurrence of the index case and the recognition of a VHF outbreak could have been prevented by a functioning VHF surveillance system in the region [4, 5]. Forest Guinea is now considered one of the high-risk zones for VHF outbreaks in sub-Sahara Africa, especially for EVD, Lassa fever, Marburg virus disease and Crimean-Congo haemorrhagic fever [6]. In particular, Lassa fever is known to be endemic in the region since the early 2000s [7]. Routine VHF screening has become an important task in public healthcare facilities in this region.

Early case detection through adequate and efficient VHF screening improves multiple outcomes: it decreases the risk of community and nosocomial transmission, reduces case fatality rates of individual patients, and reduces the occupational risk of infection for Health Care Workers (HCW) [8]. Functional VHF screening algorithms help to identify suspect cases and to detect infections using recommended laboratory tests. These activities inform measures to avoid nosocomial infection, reporting to the corresponding authorities and referral of patients to adequate treatment facilities [9, 10].

For HCW, routine screening for VHF in daily clinical practice is challenging. Clinical signs and symptoms for VHF are non-specific, making diagnosis challenging: more common gastrointestinal diseases and endemic diseases such as malaria and typhoid fever present with similar clinical symptoms as VHF [11–14]. Even though VHFs are caused by four different families of RNA viruses, they are classified together syndromically because they present similar symptoms in infected patients. Initially, symptoms usually include abrupt onset of high fever, fatigue, malaise, non-specific gastrointestinal symptoms and at a later stage multiple organ failure, shock and coma [15]. Even though haemorrhage – a striking clinical symptom - may occur in infected patients, it is infrequent and usually manifests in late stage disease [16]. Indeed, the most frequently reported symptoms of EVD patients during the 2013–2016 outbreak were fever accompanied by fatigue, headache, anorexia, vomiting, diarrhoea and abdominal pain [17]. Haemorrhagic signs are thus more specific, but not very sensitive, criteria.

Certain laboratory parameters (depending on the VHF) may indicate a VHF syndrome. But these parameters are – just like the symptoms – not specific to VHFs and can thus only offer limited clinical guidance in patients with conspicuous symptoms [18]. In the case of VHF syndrome laboratory abnormalities might include lymphocytopenia, leukocytopenia, thrombocytopenia and increased AST and ALT serum levels [15]. Confirmatory testing in suspected cases requires either RT-PCR as a direct method and/or ELISA assay as an indirect method, even though such diagnostic tools are not always available in low-resource healthcare settings [19].

In recent Ebola virus epidemics, diagnostic criteria have evolved to identify the most-at-risk patients in order that they be isolated and tested to reduce transmission. While they are primarily used in service of public health goals, these criteria can generate significant strain for the health care system when large numbers of patients subsequently found not to have EVD are isolated. Isolation practices can also spark fear and generate mistrust in the population, undermining public health efforts as patients stay away. Criteria currently in use in Guinea reflect a post-outbreak epidemiological reality and should contribute to on-going surveillance efforts aimed at rapid detection of new outbreaks. The challenge is to distinguish signal (cases) from significant background noise generated by the sheer volume of infectious cases with symptoms comparable to those of EVD, Lassa fever and other VHF.

The prevalence of cases meeting EVD and VHF suspect case definitions in “peace time” (i.e. outside of Ebola virus outbreaks) is unknown and would provide an important baseline against which a potential new epidemic could be identified. Moreover, although VHF screening is currently being revitalized in West African healthcare facilities [20], it remains unclear to what extent health care structures are capable of implementing VHF case detection and reporting in these regions, nor how such cases are currently handled.

In this study, we examined two different diagnostic criteria employed in post-epidemic Guinea; the first, the World Health Organization (WHO) criteria, were designed to be highly sensitive and for use during an active epidemic; the second, adapted from the Guinean Ministry of Health for integrated disease surveillance, were conceived for a post-epidemic setting for surveillance purposes. In this study, we ascertain the number of suspect cases identified by both sets of criteria and examine the hospital trajectories and outcomes of suspect cases generated by the second set of criteria.

## Methods

### Study setting

Our study took place at the Hôpital Régional de N’zérékoré (HRNZ), a tertiary provincial referral hospital in eastern Guinea between December 2018 and March 2019. N’zérékoré is Guinea’s second largest city with more than 300,000 inhabitants^1^ and is the capital of Guinea’s Forest region, also known as *Guinée Forestière*. This area is in the east of the country, borders Liberia and Sierra Leone, and was the epicentre of the West African epidemic. The HRNZ is the largest in the region, counting 175 beds and offering services in Internal Medicine, Surgery, Gynaecology/Obstetrics, Ophthalmology, Dental Care and a Critical Care Unit. As a regional referral centre it receives a wide variety of cases including emergency cases as well as conditions requiring specialized services. The hospital also houses one of the region’s few VHF laboratories - established in 2014 - capable of detecting Ebola virus infection in patients using RT-PCR. Other VHF such as Lassa fever can only be detected in the national laboratory for VHF in Guinea’s capital city. Blood samples of suspect cases other than EVD must be sent there for further analysis. The HRNZ thus plays a central role in routine VHF screening in the region. As an EVD diagnostic centre, the hospital is accordingly equipped with a small isolation ward for suspect and confirmed cases, which was installed during the 2014 outbreak.

HCWs at the HRNZ are expected to apply screening guidelines provided by the Guinean Ministry of Health in 2018 and adhere with WHO recommendations for integrated disease surveillance in African countries at all the hospital’s entrances [19, 21]. The Guinean guidelines define a VHF suspect case as ‘any person suffering from a fever that does not respond to any treatment for a regionally common disease’ who present ‘one of the following hemorrhagic signs: bloody diarrhea, gingival hemorrhage, purpura, conjunctival injection and hematuria’ (Table 1).

**Table 1:** Overview of different VHF suspect case definitions and clinical criteria

Definition 1	Definition 2	Definition 3	Definition 4
VHF suspect case criteria of Guinean Health Ministry	WHO EVD suspect case criteria at HRNZ Triage	VHF suspect case criteria in clinical practice at HRNZ	VHF suspect case criteria used for post-hoc screening for study
Fever not responding to treatment AND one or more of the following: bloody diarrhea, gingival hemorrhage, purpura, conjunctival injection and hematuria	Three general symptoms and fever ≥37.5 °C or history of fever. OR *any haemorrhagic sign* ^a^	Fever ≥38 °C AND any haemorrhagic sign	Fever ≥38 °C AND one or more of the following: bloody diarrhea, gingival hemorrhage, purpura, conjunctival injection and hematuria

^a^Epidemiological link and unexplained death not asked at Triage in non-outbreak setting

However, VHF suspect case criteria are applied differently at the HRNZ. All patients (not visitors) are interviewed at the hospital’s entrances with the help of the WHO EVD Triage form used since the 2013–2016 EVD epidemic [3]. This form identifies EVD suspect cases during an outbreak according to an algorithm of several criteria: general symptoms (e.g. vomit, headache, diarrhoea, nausea, etc.), pyrexia, haemorrhage, epidemiological link and unexplained death (Table 1). The triage staff may thus use this form to identify VHF suspect cases according to the Health Ministry’s definition based on symptoms, pyrexia and haemorrhage. However, in practice the EVD triage form is issued to orient patients towards different services based on symptoms reported. If a patient appears to be severely ill or injured at hospital entry, the responsible HCW classifies the patient as emergency patient without examination.

Emergency patients bypass general triage to be admitted directly to the adult or paediatric emergency room: there, they are examined, symptoms and vital signs are recorded, primary treatment is initiated and the decision to admit or not is made. It is usually emergency room physicians who actively screen for VHF suspect cases. These physicians do not apply the same VHF suspect case criteria as provided by the Ministry of Health, using a broader definition that applies to all febrile patients (≥ 38 °C) with any hemorrhagic signs. This is because in practice they often cannot determine whether the fever of patients presenting with fever upon admission is a fever that has not responded to any treatment or whether the fever has been treated at all.

For the post-hoc screening in this study, we chose a compromise between the Ministry’s definition of VHF suspect case and the clinical practice at the HRNZ. As VHF suspect case, we identified all patients who presented with fever ≥38 °C upon admission and one of the hemorrhagic signs mentioned in the ministry’s definition. This compromise was chosen as the most applicable approximation to the ministry’s definition without negating clinical practice.

### Study-design and data sources

Using a cross-sectional, retrospective study design, we analysed patient data of 4317 patients who were admitted to the hospital between December 2018 and March 2019. Patient data consisted of information compiled from admission forms at hospital triage, from admission registers of the adult and paediatric emergency rooms and from patient charts of hospitalized patients qualifying for follow-up.

Hospital triage and emergency room data served to compose patient profiles at entry and to identify patients meeting our VHF suspect case criteria used for follow-up. At triage, we recorded socio-demographic data, temperature, general symptoms and signs of unexplained bleeding of non-emergency patients upon admission. The data collected was based on WHO triage forms compiled by HRNZ triage staff and transferred into a line-list database by trained research assistants on a daily basis. Data on emergency patients who had bypassed hospital triage was collected by consulting emergency room registers which had been filled manually by emergency room physicians. This data included socio-demographic data, temperature, symptoms, preliminary diagnosis, results of rapid malaria test, emergency medication, hospitalization status and outcome (deceased/alive).

For follow-up, we screened the triage and emergency room datasets for patients who met our VHF suspect case criteria and who were also hospitalized. We retrospectively reviewed patient charts of those patients to construct their clinical trajectories, ascertaining length of stay, clinical parameters and outcomes such as survival and reason of discharge. When patient charts were incomplete, we consulted the responsible HCW for additional information.

In addition, unstructured observation was part of our participation in the daily clinical routine of the HRNZ. Insights gained from unstructured observation were used for contextual understanding and to document the management of a paediatric VHF suspect case prior to our study period.

Study data was recorded anonymously for all adult and paediatric patients admitted to the HRNZ during our study period. No data was collected for patients who entered the hospital through the hospital’s HIV clinic (*n* = 142), or who bypassed triage or the emergency room (HRNZ staff have estimated around 90 individuals that bypassed, but the precise number is unknown due to absence of records). Such patients were able to bypass triage e.g. because of personal relationships to hospital staff. Thus, an estimated 94.9% (4317/4549) of all patients admitted to the HRNZ during our study period were included in our study.

### Data analysis

We stratified patients into three subgroups: triage patients, adult emergency patients and paediatric emergency patients. Since HRNZ entry points do not consistently categorize patients by age thresholds into adult and paediatric, the study design follows the hospital’s practical functioning: only emergency patients are treated by separate paediatric and adult wards but age ranges overlap since surgical paediatric emergency patients are treated by the emergency service for adults. Our stratified dataset reflects this practice.

Patient symptoms and diagnosis were coded according to the International Classification of Primary Care, 2nd edition (ICPC-2). All data was recorded and coded with Microsoft Excel 16 and descriptive analysis was executed with IBM SPSS 25 [22]. Frequency distribution tables were constructed. Because of extreme outliers in age, we used non-parametric median and interquartile range (IQR) to describe the age of admitted patients.

### Ethical considerations

Ethical approval for this study was granted by the Guinean Ethics Committee for Research in Health (opinion number 103/CNERS/18) and the Ethics Committee for Medical Research at the Ludwig-Maximilians-Universität (LMU), Munich, Germany (opinion number 18–834). Before its implementation, the study was presented to the regional health authorities and the HRNZ directorate who both consented to its implementation. As data was collected as part of routine clinical practice, and in the further analysis data presentation for the overall population is presented in an aggregate manner, no informed consent was asked from patients upon admission for the cross-sectional data collection. Informed consent to collect data from patient files was obtained from the post-hoc screened hospitalized patients as well as the parents of paediatric suspect cases.

## Results

### Patient profiles upon hospital admission

In total, 4317 patients were admitted through triage or the emergency wards during our study period (Table 2). Median age was 27 years (IQR 11–45). 50.8% of patients were female, and the majority of patients (78.2%) resided in an urban area. Slightly more than half of all the patients (51.1%) were admitted to gynaecological, surgical, paediatric or internal medical services. Fever was the most frequently self-reported symptom upon admission (19.9%), especially amongst paediatric patients where 86.2% of parents claimed that their child was suffering from fever. 9.5% of all patients had a measured body temperature ≥ 38 °C and all but one of those febrile patients were admitted through the emergency wards. Abdominal pain (17.7%) and weakness/tiredness (16.4%) were the second and third most frequently reported symptoms. In addition, other general and gastrointestinal symptoms, such as headache, loss of appetite, nausea/vomit and diarrhoea were common complaints.

**Table 2:** Patient profiles upon admission at different entry points

	Triage	Adult Emergency Room	Paediatric Emergency Room	Total
Total Number of Patients N	2616	1178	523	4317
General Characteristics				
Median Age – years (IQR)	29 (16–45)	35 (23–55)	1 (1–4)	27 (11–45)
Male Sex – n/N (%)	1134/2616 (43.3)	691/1178 (58.7)	296/523 (56.6)	2121/4317 (49.1)
Female Sex – n/N (%)	1482/2616 (56.7)	484/1178 (41.1)	227/523 (43.4)	2193/4317 (50.8)
Sex not registered – n/N (%)	0/2616 (0.0)	3/1178 (0.3)	0/523 (0.0)	3/4317 (0.1)
Residence in urban area – n/N (%)	2127/2616 (81.3)	863/1178 (73.3)	385/523 (73.6)	3375/4317 (78.2)
Residence in rural area – n/N (%)	487/2616 (18.6)	183/1178 (15.5)	125/523 (23.9)	795/4317 (18.4)
Residence not registered – n/N (%)	2/2616 (0.1)	132/1178 (11.2)	13/523 (2.5)	147/4317 (3.4)
Most frequent symptoms	Abdominal Pain, Headache, Fever	Tiredness/ Weakness, Abdominal Pain, Headache	Fever, Tiredness/ Weakness, Loss of Appetite	Fever, Abdominal Pain, Tiredness/ Weakness
Symptoms				
Self-reported Fever – n/N (%)	212/2616 (8.1)	195/1178 (16.6)	451/523 (86.2)	858/4317 (19.9)
Abdominal Pain – n/N (%)	507/2616 (19.4)	211/1178 (17.9)	46/523 (8.8)	764/4317 (17.7)
Tiredness/Weakness – n/N (%)	56/2616 (2.1)	282/1178 (23.9)	371/523 (70.9)	709/4317 (16.4)
Loss of appetite – n/N (%)	146/2616 (5.6)	176/1178 (14.9)	274/523 (52.4)	596/4317 (13.8)
Headache – n/N (%)	371/2616 (14.2)	199/1178 (16.9)	23/523 (4.4)	593/4317 (13.7)
Nausea/Vomit – n/N (%)	189/2616 (7.2)	132/1178 (11.2)	254/523 (48.6)	575/4317 (13.3)
Documented Fever ≥38 °C – n/N (%)	1/2616 (0.0)	182/1178 (15.4)	229/523 (43.8)	412/4317 (9.5)
Diarrhoea – n/N (%)	66/2616 (2.5)	83/1178 (7.0)	159/523 (30.4)	308/4317 (7.1)
Vertigo/Dizziness – n/N (%)	0/2616 (0.0)	142/1178 (12.2)	1/523 (0.2)	143/4317 (3.3)
Myalgia/Joint pain – n/N (%)	77/2616 (2.9)	13/1178 (1.1)	0/523 (0.0)	90/4317 (2.1)
Bloody stool/Melena – n/N (%)	6/2616 (0.2)	16/1178 (1.4)	5/523 (1.0)	27/4317 (0.6)
Other unexplained bleeding – n/N (%)	22/2616 (0.8)	0/1178 (0.0)	0/523 (0.0)	22/4317 (0.5)
Hematemesis – n/N (%)	0/2616 (0.0)	15/1178 (1.3)	0/523 (0.0)	15/4317 (0.3)
Epistaxis – n/N (%)	7/2616 (0.3)	3/1178 (0.3)	0/523 (0.0)	10/4317 (0.2)
Haematuria – n/N (%)	0/2616 (0.0)	4/1178 (0.3)	3/523 (0.6)	7/4317 (0.2)
Haemoptysis – n/N (%)	0/2616 (0.0)	3/1178 (0.3)	0/523 (0.0)	3/4317 (0.1)
Red eyes/Conjunctivitis – n/N (%)	1/2616 (0.0)	0/1178 (0.0)	0/523 (0.0)	1/4317 (0.0)
Hospitalization Status				
Outpatient – n/N (%)	1524/2616 (58.3)	529/1178 (44.9)	57/523 (10.9)	2110/4317 (48.9)
Hospitalized in Surgery– n/N (%)	205/2616 (7.8)	209/1178 (17.7)	0/523 (0.0)	414/4317 (9.6)
Hospitalized in Internal Medicine– n/N (%)	22/2616 (8.4)	440/1178 (37.4)	0/523 (0.0)	462/4317 (10.7)
Hospitalized in Paediatrics – n/N (%)	363/2616 (13.9)	0/1178 (0.0)	466/523 (89.1)	829/4317 (19.2)
Hospitalized in Gynaecology – n/N (%)	502/2616 (19.2)	0/1178 (0.0)	0/523 (0.0)	502/4317 (11.6)

Haemorrhagic signs were less frequent. In total, 27 patients (0.6%) reported bloody stool or melena, 15 patients (0.3%) vomiting blood, ten patients (0.2%) a nose bleed, seven patients (0.2%) haematuria, three patients (0.1%) haemoptysis and one patient conjunctivitis. An additional 22 patients (0.5%) showed other haemorrhagic signs upon admission. None presented with purpura or gingival bleeding.

A total of twelve patients (0.3%) met the study-specific, non-outbreak VHF suspect case criteria, 18 patients (0.4%) met non-outbreak VHF suspect case criteria used in clinical practice and a total of 686 patients (15.9%) met EVD outbreak criteria (Table 3). Ten of the patients identified by study-specific criteria were admitted through the adult emergency room, of which seven were hospitalized in Internal Medicine, two patients were discharged after emergency treatment and one patient died during emergency treatment. In the paediatric emergency room, two patients met the VHF suspect case definition and both were hospitalized in the paediatric service. Of the 18 patients identified by non-outbreak criteria used in clinical practice, none was treated as VHF suspect case or tested for VHF.

**Table 3:** Frequency of patients meeting different VHF suspect case definitions upon admission

	Triage	Adult Emergency Room	Pediatric Emergency Room	Total
Total N	2616	1178	523	4317
Three general symptoms (e.g. vomiting, headache, diarrhoea, nausea, etc.) and fever ≥37.5 °C or history of fever (Definition 2) – n/N (%)	212/2616 (8.1)	153/1178 (13.0)	235/523 (44.9)	600/4317 (13.9)
Any haemorrhagic sign (Definition 2) – n/N (%)	36/2616 (1.4)	42/1178 (3.6)	8/1178 (1.5)	86/4317 (2.0)
Fever ≥38.0 °C and any haemorrhagic sign (Definition 3) – n/N (%)	0/2616 (0.0)	16/1178 (1.4)	2/523 (0.4)	18/4317 (0.4)
Fever ≥38.0 °C and bloody diarrhea, gingival hemorrhage, purpura, conjunctival injection, hematuria (Definition 4) – n/N (%)	0/2616 (0.0)	10/1178 (0.8)	2/523 (0.4)	12/4317 (0.3)

Six hundred patients (13.9%) fulfilled WHO EVD suspect case criteria upon admission, based on three general symptoms (e.g. vomiting, headache, diarrhoea, nausea, etc.) and fever ≥37.5 °C or a history of fever (Table 3). Another 86 patients (2.0%) presented with unexplained bleeding and thus also met WHO EVD suspect case criteria.

### Clinical trajectories of hospitalized patients meeting study-specific VHF suspect case criteria

**Fig. 1:**
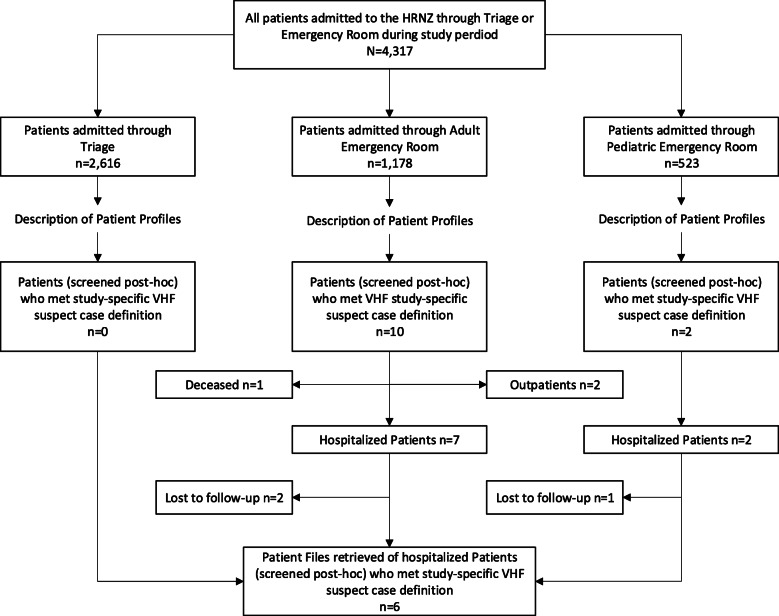
Patient admission at different wards and post-hoc VHF suspect case screening: see attached file

Nine of the twelve patients who met study-specific VHF suspect case criteria were hospitalized. Three of those patients were lost to follow-up because their patient files could not be found. Five patient files were retrieved from the Internal Medicine ward and one patient file was retrieved from the Paediatric ward (Fig. 1). None of the patients who fulfilled VHF suspect criteria were treated as a VHF suspect case or tested upon admission or during hospitalization. During the study period, no VHF suspect case was reported by the HRNZ to the authorities.

Three of the six post-hoc screened patients came from an urban area, and three from a rural area (Table 4). Next to fever ≥38 °C, all but one of the patients presented with bloody diarrhoea as a haemorrhagic sign. Preliminary diagnoses for most patients were either malaria, gastrointestinal infection/ typhoid fever or gastro-duodenal ulcer. All but one patient received antibiotics upon admission. Four patients tested negative for malaria with a rapid antigen test. The paediatric patient tested negative for malaria by thick blood smear after hospitalization. The remaining two patients were not tested for malaria. Haemoglobin and blood sugar levels were measured for all patients at the emergency room. However, despite determining the blood type of some patients for administering blood transfusions, no other laboratory parameters were obtained during hospitalization for any of the patients: in five cases, Widal test was demanded by the treating physician, in two cases, stool parasitology, in one case a chest X-ray and in one case a thick blood smear. Prescribed tests were not performed for the following reasons: no laboratory requisitions were given to the patient, the patient did not go to the laboratory for testing, or the patient could not afford the laboratory fees. No other parameters such as blood cell count or transaminase levels were demanded by the treating physician during hospitalization of any of the six patients.

**Table 4:** Diagnostic trajectories of hospitalized patients meeting study-specific VHF suspect case criteria

	Patient A	Patient B	Patient C	Patient D	Patient E	Patient F
Residence	Rural	Rural	Rural	Urban	Urban	Urban
Symptoms upon admission	Bloody Diarrhoea, Oedema, Weight Loss, Weakness	Bloody Diarrhoea, Weight Loss, Cough, Chest Pain	Bloody Diarrhoea, Vomit, Epigastric Pain, Weakness	Haematuria, Fever, Weakness, Epigastric Pain, Weight Loss	Bloody Diarrhoea, Hematemesis, Dizziness, Fever, Weakness	Bloody Diarrhoea, Hematemesis, Dizziness, Fever, Headache, Epigastric Pain
Measured Temperature °C upon admission	38.5	39.5	39.3	39.0	38.5	38.2
Rapid Malaria Test upon admission	Negative	Not tested	Not tested	Negative	Negative	Negative
Preliminary Diagnosis upon admission	Malaria, Gastrointestinal Infection/Typhoid Fever	HIV/AIDS, Pulmonary Tuberculosis	Gastrointestinal Infection/Typhoid Fever, Gastro-duodenal Ulcer	Malaria, Gastrointestinal Infection/Typhoid Fever	Gastrointestinal Infection/Typhoid Fever, Gastro-duodenal Ulcer, Anaemia	Gastro-duodenal Ulcer, Anaemia
Emergency Treatment	Ampicillin, Metronidazole, Zinc	Ampicillin, Ringer’s Lactate	Ampicillin, Ceftriaxone, Omeprazole, Paracetamol, Ringer Lactate	Ceftriaxone, Omeprazole, Paracetamol	none	Ampicillin, Omeprazole, Paracetamol, Ringer Lactate
Service	Paediatrics	Internal Medicine	Internal Medicine	Internal Medicine	Internal Medicine	Internal Medicine
Amount of Days Hospitalized	2	3	5	3	4	5
Diagnostic Tests demanded during Hospitalization	Haemoglobin, Glycaemia, Widal, Thick Blood Smear, Blood Type	Haemoglobin, Glycaemia, Chest X-Ray	Haemoglobin, Glycaemia, Widal, Stool Parasitology	Haemoglobin, Glycaemia, Widal	Haemoglobin, Glycaemia, Widal, Thick Blood Smear, Blood Type	Haemoglobin, Glycaemia, Widal, Stool Parasitology, Blood Type
Diagnostic Results obtained	Haemoglobin, Glycaemia, Thick Blood Smear (negative), Blood Type	Haemoglobin, Glycaemia	Haemoglobin, Glycaemia	Haemoglobin, Glycaemia	Haemoglobin, Glycaemia, Blood Type	Haemoglobin, Glycaemia, Blood Type
Diagnostic Results not obtained	Widal	Chest X-Ray	Widal, Stool Parasitology	Widal	Widal, Thick Blood Smear	Widal, Stool Parasitology
Definite Diagnosis	None	HIV/AIDS (not Laboratory confirmed)	Gastrointestinal Infection, Cholecystitis	None	Gastrointestinal Infection/Typhoid Fever, Anaemia	Gastro-duodenal Ulcer, Anaemia
Discharge Reason	Self-discharge	Self-discharge	Improved Health	Self-discharge	Improved Health	Improved Health

Including haemoglobin and glycaemia levels obtained in the emergency room, a total of 25 laboratory parameters were requested for the six patients from the moment of admission until discharge, of which nine (36.0%) were not obtained. Without the two emergency parameters routinely measured for any emergency patient upon admission, a total of 13 laboratory parameters were requested for the six patients during their hospitalization of which nine (69.2%) were not obtained.

Patients’ diagnoses were thus based on their clinical presentation. One patient was diagnosed with HIV/AIDS exclusively on clinical grounds with no corresponding laboratory confirmation even though the HRNZ provides HIV testing free of charge to all patients. Two patients were diagnosed with a gastrointestinal infection or typhoid fever or cholecystitis. One patient was presumptively diagnosed with gastro-duodenal ulcer on clinical grounds but there was no possibility at the HRNZ to perform endoscopy to confirm diagnosis. Two patients did not receive any diagnosis before discharge.

In regard to the discharge diagnosis, three patients were discharged with improved health and three patients discharged themselves against medical advice. Length of hospital stay for patients was between two and five days.

### Description of a recognized VHF suspect case

One week before we initiated cross-sectional data collection, the HRNZ reported a suspect case to the regional health authorities: a paediatric patient had been referred to the HRNZ by a rural health centre presenting with fever and epistaxis. At that health centre, the patient received Ampicillin for several days prior to referral but the fever had persisted and the epistaxis raised the supervising HCW’s attention. The referring HCW was criticized by other HCW and the patient’s parents for identifying the patient as VHF suspect case and initiating the corresponding measures. Malaria had been ruled out and the patient was referred to the HRNZ as VHF suspect case. Upon admission at the HRNZ the patient’s haemorrhagic signs were not recorded at the emergency room and the patient was hospitalized in the paediatric service without further precautions. The initial diagnosis upon admission was pulmonary infection. Malaria was again ruled out using rapid malaria test and thick blood smear, and Ampicillin was continued. No additional laboratory parameters were obtained. Two days after admission, as the symptoms persisted, the patient was isolated and tested negative for EVD at the HRNZ laboratory. The patient was not tested for any other VHF and no blood samples were sent to the national VHF laboratory for further analysis. To implement isolation measures, three HCWs had to convince the patient’s father of their necessity, and eventually had to prevent him from discharging his son against medical advice. The patient’s condition improved after several days of hospitalization and he was discharged with the diagnosis of bacterial pneumonia.

## Discussion

To our knowledge, this is the first study in Forest Guinea to report the facility-based period prevalence of VHF suspect cases at a tertiary referral hospital after the 2013–2016 EVD outbreak. We found that twelve patients in three months met study-specific VHF suspect case definition none of those patients were treated as VHF suspect case and no patient was tested for EVD or other VHF during our study period. Our study suggests that the identification of suspect cases seems difficult in clinical practice because of the non-application of any VHF suspect case criteria and the low use of diagnostic capacities to rule out more likely causes for VHF-like symptoms.

Many patients were admitted to the HRNZ during the study period with general or gastrointestinal symptoms and/or fever. This finding is not surprising, keeping in mind the frequency of certain tropical diseases and gastrointestinal infections with such symptoms in the region [23]. However, in an EVD outbreak situation, 686 patients would have been considered suspect cases due to the application of different, more sensitive suspect case criteria. This volume would become a significant challenge to the hospital’s capacity to manage, isolate and refer all potentially infectious patients. Our study suggests that the ability of the hospital to perform adequate triage and diagnosis of a fairly low volume of VHF suspect cases in a non-outbreak setting is already impaired: the hospital did not recognize any patient as VHF suspect case regardless of the criteria used upon admission. Our assumptions with regards to why hospitals fail to identify VHF suspect cases are based on personal observations undertaken during the study period as well as various informal conversations with hospital staff. Hospital staff face several interconnected difficulties in screening VHF suspect cases. Our observations discussed below largely resonate with prior studies and reports regarding the role of trust in and within healthcare institutions during Ebola virus epidemics [24–27]. We observed that the Ebola virus epidemic has generated a profound unease with VHF in general and EVD in particular in the clinical context. During data collection, while hospital staff were aware of technical details such as symptoms and treatment of EVD, they were reluctant to label a patient a “suspect case”. Identifying a VHF suspect case could result negative consequences and the staff appeared to be reluctant to take responsibility for “bringing back” the disease to their clinical reality by identifying a VHF suspect. Further, by avoiding the correct application of VHF suspect case criteria, hospital staff sidestepped negative consequences, e.g. the strong and at times violent reactions by patients if these were characterised as a VHF suspect case. Each VHF suspect case was believed to have the potential to disrupt the hospital’s functioning since word of a suspect case would spread quickly amongst staff, patients and visitors leading to general panic. We thus believe that in post-Ebola times, understanding the psychosocial effects of the EVD epidemic on clinical staff and patients is of utmost importance in any attempt to improve VHF screening in daily clinical practice of public healthcare facilities in Guinea and beyond.

We identified non-use and absence of certain laboratory diagnostics as another challenge in VHF screening. According to national recommendations developed by Guinean authorities in collaboration with the World Health Organization and other partners, identifying VHF in clinical practice outside epidemics requires ruling out other, more likely causes for a patient’s symptoms such as malaria, meningitis, leptospirosis, septic shock and HIV [28]. Affordable laboratory diagnostic tools to perform complete blood count, blood and urine cultures for those and other common regional infectious diseases are thus a central element in the task of VHF screening. The low availability and use of certain laboratory parameters to establish causative pathogens in potentially infectious patients observed in our study is due to multiple factors which are generally a consequence of known problems in the Guinean healthcare system: chronic underfunding, pay-as-you-go user fees and low levels of hospital staff training [29]. Improvements made on this level could not only facilitate screening for VHF with all benefits, but also guide adequate treatment of patients suffering from common infectious diseases, improving their clinical outcome.

By describing the numbers of non-outbreak VHF suspect cases in the observed population and EVD outbreak criteria (*n* = 12 or *n* = 18 vs. *n* = 686), our study identifies a weakness in the VHF suspect case definition for routine screening: haemorrhagic signs are an infrequent clinical feature of EVD and other VHF, but the authors of non-outbreak suspect case criteria insist on their presence. This fact is mainly responsible for the large difference in the number of suspect cases identified by non-outbreak vs. outbreak criteria (n = 12 or n = 18 vs. n = 686). While the insistence on the presence of haemorrhagic signs reduces the procedural sensitivity and correspondingly VHF suspect case numbers to a manageable amount, the current definition used by the Guinean Ministry - adapted from WHO recommendations for integrated disease surveillance – may risk to miss signal cases who may not present with signs of haemorrhage.

### Limitations

Firstly, our study has been implemented at one tertiary referral hospital in Guinea only and the findings are thus of limited generalizability for similar healthcare structures. It needs to be verified whether other structures face the same or similar challenges with routine VHF screening. Secondly, our suggested prevalence regarding VHF suspect cases should be read with caution. The suspect case definition for post-hoc screening used in the study is not fully identical to the Guinean Health Ministry’s definition or the suspect case criteria HRNZ staff claimed to be using in real practice. Especially the described quality of a patient’s fever within the Ministry’s definition, that is, persistence of fever despite treatment, made it impossible to apply identical suspect case criteria. Nonetheless, the criteria used in our study for post-hoc screening is the best possible compromise between official protocol and clinical practice as it also encompasses patients theoretically meeting the more generous, i.e. sensitive definition of HCWs in their clinical practice: all patients identified as VHF suspect cases by our post-hoc screening algorithm (*n* = 12) should have been identified by HCW by applying the suspect case criteria currently in force (*n* = 18). Our findings can thus only provide for a rough estimation of the continuous emergence of VHF suspect cases. More precise management depends on how suspect case criteria are applied in clinical practice. However, this limitation does not apply to the observational data challenges in the VHF screening we identified.

Thirdly, cross-sectional studies only allow for a snapshot in time, and estimations of the influx of VHF suspect cases for longer periods may encounter considerable variations over time. In general, hospital admission numbers as well as patient symptoms may vary throughout the year, depending on the season. Certain seasons provide the farming population and merchants with more means for healthcare spending. Similarly, the start of the rainy season in March usually increases the amount of malaria cases in the region and thus the number of febrile patients with symptoms similar to VHF.

## Conclusions

West Africa in general and Forest Guinea in particular are at risk for future VHF outbreaks. Tertiary referral hospitals are key, as they receive potentially infectious patients with conspicuous symptoms and often offer better diagnostic capacities than rural health centres. Our study suggests that the number of VHF suspect cases at the largest tertiary referral hospital in Forest Guinea is fairly low when using a suspect case definition for routine surveillance in a non-outbreak setting. Nevertheless, even low numbers of suspect cases go unrecognized because suspect case criteria are not rigorously applied by clinical staff. Furthermore, diagnostic tools to identify more probable causes than VHF in patients such as malaria, meningitis or septic shock are rarely used. In the hypothetical case of another EVD outbreak in which WHO suspect case criteria were used, suspect case numbers would highly increase due to the large quantity of febrile patients with general symptoms. Managing such a high volume of suspect cases in an outbreak setting would become a serious challenge to tertiary referral hospitals, considering the fact that already a small amount of suspect cases in “peace time” goes unrecognized. The resulting isolation measures imposed on such large numbers of suspect cases might reproduce the 2013–2016 EVD outbreak experience as an “epidemic of mistrust”. Current and future efforts to establish routine screening for VHF in West Africa should take these findings into account.

## Data Availability

Due to Guinean national regulations, patient-based datasets cannot be made freely accessible. Dataset can be provided, however, upon well-reasoned request and upon clearance by the involved Guinean authorities. Requests should be addressed to the corresponding author.

## Abbreviations

EVD: Ebola Virus Disease
HCW: Health Care Worker
HRNZ: Hôpital Régional de N’Zérékoré
RT-PCR: Reverse Transcriptase Polymerase Chain Reaction
VHF: Viral Haemorrhagic Fever
WHO: World Health Organization

## Footnotes

1 Obtained from the N’zérékoré city hall in January, 2019.
